# Dietary patterns and cardio-metabolic risk in a population of Guatemalan young adults

**DOI:** 10.1186/s40795-017-0188-5

**Published:** 2017-07-28

**Authors:** Nicole D. Ford, Lindsay M. Jaacks, Reynaldo Martorell, Neil K. Mehta, Cria G. Perrine, Manuel Ramirez-Zea, Aryeh D. Stein

**Affiliations:** 1 Nutrition and Health Sciences Program, Laney Graduate School, Emory University, Atlanta, GA USA; 2 Department of Global Health and Population, Harvard T.H. Chan School of Public Health, Harvard University, Boston, MA USA; 3 Hubert Department of Global Health, Rollins School of Public Health, Emory University, Atlanta, GA USA; 4 Department of Health Management and Policy, University of Michigan, Ann Arbor, MI USA; 5 Division of Nutrition, Physical Activity, and Obesity, U.S. Centers for Disease Control and Prevention, Atlanta, GA USA; 6 INCAP Research Center for the Prevention of Chronic Diseases (CIIPEC), Institute of Nutrition of Central America and Panama, Guatemala City, Guatemala; 7 1518 Clifton Road NE, CNR 7007, Atlanta, GA 30033 USA

**Keywords:** Dietary pattern, Cardio-metabolic disease, Guatemala, Nutrition transition

## Abstract

**Background:**

Latin America is facing an increasing burden of nutrition-related non-communicable disease. Little is known about dietary patterns in Guatemalan adults and how dietary patterns are associated with cardio-metabolic disease (CMD) risk.

**Methods:**

This analysis is based on data from a 2002–04 follow-up study of the INCAP Nutrition Supplementation Trial Longitudinal Cohort. Diet data were collected using a validated, semi-quantitative food frequency questionnaire. We derived dietary patterns using principal components analysis. CMD risk was assessed by anthropometry (body mass index, waist circumference), biochemistry (fasting blood glucose and lipids), and clinical (blood pressure) measures. We used sex-stratified multivariable log binomial models to test associations between dietary pattern tertile and CMD risk factors. The sample included 1428 participants (681 men and 747 women) ages 25–43 years.

**Results:**

We derived three dietary patterns (traditional, meat-based modern, and starch-based modern), collectively explaining 24.2% of variance in the diet. Dietary patterns were not associated with most CMD risk factors; however, higher starch-based modern tertiles were associated with increased prevalence of low highdensity lipoprotein cholesterol (HDL-c) in men (Prevalence Ratio (PR) 1.17, 95% Confidence Interval (CI) 1.01, 1.20 for tertile 2; PR 1.20, 95% CI 1.00, 1.44 for tertile 3; p trend 0.04). Higher traditional tertiles were associated with increased prevalence of abdominal obesity in women (PR 1.24, 95% CI 1.07, 1.43 for tertile 2; PR 1.19, 95% CI 1.02, 1.39 for tertile 3; p trend 0.02) but marginally significant reduced prevalence of low HDL-c in men (PR 0.88, 95% CI 0.76, 1.00 for tertile 2; PR 0.85, 95% CI 0.72, 1.00 for tertile 3; p trend 0.05).

**Conclusion:**

Our findings suggest the presence of two ‘modern' dietary patterns in Guatemala – one of which was associated with increased prevalence of low HDL-c in men. The association between the traditional dietary pattern and some CMD risk factors may vary by sex.

**Electronic supplementary material:**

The online version of this article (doi:10.1186/s40795-017-0188-5) contains supplementary material, which is available to authorized users.

## Background

Latin America has experienced large increases in non-communicable diseases (NCDs) over the past 20 years, which now account for 34% of deaths in the region [1]. These increases are a direct result of demographic, social, and economic changes influencing dietary and physical activity patterns, among other factors [2]. The nutrition transition is marked by changes in diet from staple foods to high-fat, high-sugar, processed foods [3]. Despite documented changes to the food environment in Guatemala, such as doubling of the number of supermarkets from 1990 to 2008 [4] and increased imports of processed foods [5], the extent to which Guatemalans have adopted modern diets is unclear. To date, little has been published about individual diets in Guatemala [6–9].

Furthermore, there is little information about how Guatemalan dietary habits influence NCD risk. One previous study of diet scores and cardio-metabolic disease (CMD) risk found that neither score-based indices of diet quality nor single nutrients were consistently associated with CMD risk factors [10–12]; however, individuals with identical diet scores can have diverse consumption patterns [13]. Data-driven dietary pattern analysis goes beyond intake and adequacy of individual nutrients and attempts to characterize dietary behavior and link patterns of consumption of foods and beverages to health outcomes, including adult anthropometry, metabolic syndrome, and diabetes [14–16]. Principal components analysis (PCA) is a commonly used method in nutritional epidemiology that aims to explain variation in food intake. PCA has advantages over alternative data-driven methods, such as reduced rank regression, which could identify dietary patterns not actually consumed in the study population [17]. Because diet is a major modifiable determinant of NCD risk [18], understanding how dietary patterns are associated with CMD risk is important for informing public health strategies.

The objectives of this study were: (a) to characterize dietary patterns using PCA; and (b) to examine the association between dietary patterns and CMD risk in a population of Guatemalan adults.

## Methods

### Study population

Individuals in this study were born in four villages in *El Progreso* department in Guatemala from 1962 to 1977 and were participants in the Institute of Nutrition of Central America and Panama (INCAP) Nutrition Supplementation Trial Longitudinal Study cohort [19]. This analysis was based on the 2002–04 follow-up. Full details of the original nutrition supplementation trial and its follow-up waves are published elsewhere [20]. Of the 2392 participants in the original study, 1855 (77.5%) were alive and living in Guatemala for the 2002–04 follow-up, of which 1571 (85%) consented to participate and provided at least some data. Data were collected in four sessions over the course of 1 year. Dietary data were acquired during one of these sessions. We obtained dietary data from 1488 (62.2% of the original cohort) adults ages 25–43 years. The cohort members who consented to participate and who provided at least partial information but were missing dietary data (*n* = 83) were more likely to be male.

Participants were excluded from analyses if they had total energy intake that exceeded 3 SD from the median total energy intake (2544 kcal) (*n* = 19) or if they were pregnant or within 6 months post-partum (*n* = 41). The final analytic sample included 1428 participants (681 men and 747 women); of these 1283 had anthropometric measures, 1141 had blood data, and 1355 had blood pressure.

All data collection followed protocols that were approved by the institutional review boards of Emory University (Atlanta, GA) and INCAP (Guatemala City, Guatemala). All participants gave written informed consent.

### Assessment of dietary intake

Data on typical dietary intake were collected using a validated, semi-quantitative 52-item food frequency questionnaire (FFQ) [21]. Participants were asked about their consumption over the last 3 months of traditional and “transitional” foods such as pizza and hamburgers. The FFQ also included open-ended sections for fruits and vegetables to capture potential seasonality in consumption of these foods. The frequency categories were never/rarely, daily, days per week, and days per month. Portion sizes were determined by selecting from a range of serving sizes (e.g. small, medium, or large tortillas; three different serving spoons) or standard serving units (e.g. a medium-sized apple). We converted frequency of consumption for each food to servings per day and then summed the servings for food items. We estimated nutrient intake using the INCAP nutrient database, which is based on foods commonly consumed in Guatemala, supplemented with data from the United States (U.S.) Department of Agriculture Nutrient Database, assuming standard weights based on the reported serving sizes. Servings of food items were converted to grams per day consumed.

### Derivation of dietary patterns

We categorized food items into 27 mutually exclusive food groups adapted from food group categories developed in Mexico (Additional file 1: Table S1) [22]. Because <10% of participants reported any consumption of “Nuts” and “Whole Grains”, we removed these food groups to improve statistical robustness [23, 24]. We also removed “Coffee” from the analyses owing to its high correlation with “Sugar Added to Coffee” (R^2^ = 0.89) which led to instability in the factor solutions. Because coffee contributes few calories per mL relative to each gram of sugar, we chose to retain “Sugar Added to Coffee” in analyses.

To derive the dietary patterns, we used PCA with orthogonal rotation of total grams of food groups consumed. We used scree plots, eigenvalues >1.4, minimum absolute factor loading of 0.35, and the interpretability of factors to guide the final selection of dietary patterns [25]. We also explored dietary patterns for men and women separately; however, the patterns (i.e. food groups and factor loadings) were largely consistent, so we conducted the final PCA on the entire sample. Individual dietary pattern scores were calculated by multiplying food group factor loadings by individual intake of the food group (in grams) and then summing for each dietary pattern [25]. Due to large differences in energy intake by sex, we categorized the resulting dietary pattern scores into sex-specific tertiles. To assess the stability of the factor solutions, we randomly split the study sample and repeated the analyses [26–28].

### Assessment of anthropometric, biochemical, and clinical measures

Trained field workers collected all anthropometric measurements. Height and waist circumference were measured to the nearest 0.1 cm and weight to the nearest 0.01 kg. All measurements were taken in duplicate; if the difference exceeded 0.5 kg for weight or 0.5 cm for height or 1.0 cm for waist circumference, a third measurement was taken and the average of the two closest measurements was used. Body mass index (BMI) was calculated as weight (kg) divided by height-squared (m^2^) and was classified according to current National Heart, Lung, and Blood Institute (NHLBI) guidelines: underweight/normal (<25.0 kg/m^2^) and overweight/obese (≥25.0 kg/m^2^) [29]. Abdominal obesity was defined according to the NHLBI’s 2005 National Cholesterol Education Program Adult Treatment Panel III (NCEP ATP III) cut-point of waist circumference of ≥102 cm for men and ≥88 cm for women [30].

For blood lipids and glucose, trained field staff drew fasted (>9 h) capillary blood samples. Lipids (total cholesterol, high density lipoprotein cholesterol [HDL-c], and triglycerides) and glucose concentrations were measured by enzymatic peroxidase dry chemistry methods (Cholestech LDX System, Hayward, CA, USA). Elevated triglycerides were defined as triglycerides ≥150 mg/dL [30]. Low HDL-c was defined as <40 mg/dL for men and <50 mg/dL for women [30]. Dysglycemia was defined as having either impaired fasting glucose (fasting blood glucose 100–125 mg/dL) or diabetes (fasting blood glucose ≥126 mg/dL and/or self-reported diabetes and/or reported diabetes medication use).

Seated blood pressure was measured by a physician three times at three-minute intervals on the left arm resting on a table at heart level using a digital blood pressure monitor (Omron, Schaumburg, IL, USA) after a 5 minute rest [31]. Three cuff sizes were available and selected based on participant arm circumference. If systolic or diastolic blood pressure measurements differed by >10 mmHg, then a fourth measure was taken. Otherwise, the second and third measurements were recorded and the average of these two measures used. Medication use for hypertension was ascertained by interview.

Metabolic syndrome was defined according to 2005 NCEP ATP III diagnostic criteria based on presence of at least three of the following: abdominal obesity (waist circumference ≥ 102 cm for men; ≥88 cm for women); fasting glucose ≥100 mg/dL or medication; triglycerides ≥150 mg/dL or medication; HDL-c < 40 mg/dL in men or <50 mg/dL in women; and blood pressure > 130 mmHg systolic, >85 mmHg diastolic, and/or medication use [30].

### Assessment of covariates

Data on lifestyle and socioeconomic factors were collected by interview. Potential covariates were identified a priori and include age, socioeconomic status (SES), urban residence, smoking, multivitamin use, and physical activity. SES was a cumulative score developed from PCA of participant household characteristics and consumer durable goods [32]. Urban residence was based on residence location, neighborhood, and amenities [11]. Current smoking and daily multivitamin intake were classified as yes or no. Average physical activity was ascertained using a physical activity questionnaire asking about the frequency and duration of activities performed on a typical day over the preceding year [33]. Participant physical activity level (PAL) was calculated by averaging metabolic equivalents (MET) per hour over 24 h. Participants were classified as physically inactive if their PAL was <1.7 – the minimum level recommended to prevent obesity [34].

### Statistical analyses

To assess differences in sociodemographic, health characteristics, and in dietary factors such as percent of energy from macronutrients across dietary pattern tertiles, we used Mantel-Haenszel chi-square tests for categorical variables and Spearman rank tests for continuous variables. To characterize the relationship between dietary pattern and CMD risk factors, we used sex-stratified log binomial models to regress CMD risk outcomes on dietary pattern score tertiles. Due to the high prevalence of most of the CMD risk factors in our population, we used log-binomial models to estimate relative risk. Model 1 controlled for age. Model 2 additionally controlled for total energy intake to account for absolute differences in food intake [35]. Model 3 additionally controlled for other covariates (urban residence, SES, and multivitamin use). We also controlled for smoking and physical inactivity in Model 3 in men; too few women smoked or were physically active to include these variables in the model. Each dietary pattern was modeled separately. We also assessed effect modification by stratifying the data by urban residence and comparing stratum-specific and pooled prevalence ratio estimates [12]. All models converged normally.

Statistical significance was set a priori at *P* < 0.05. For statistical interactions, *P* < 0.10 was considered significant. All *P*-values were two-sided. All analyses were performed using SAS v.9.4 (SAS Institute, Cary, NC, USA).

## Results

We identified three dietary patterns which we label as meat-based modern, starch-based modern, and traditional; these dietary patterns collectively explained 24.2% of the dietary variance (Tables 1 and 2). The meat-based modern pattern was characterized by consumption of salty snacks, processed and fried meats, sweets/candies, alcohol, soda, traditional Guatemalan foods (e.g. tamales and tacos), and transitional foods (e.g. hamburgers and pizza). The starch-based modern pattern was characterized by consumption of refined grains, starchy and non-starchy vegetables, fried starches, juices, fruit, oils/fats, giblets, and packaged soups. The traditional pattern was characterized by consumption of corn tortillas, beans, sugar added to coffee, and was negatively associated with consumption of dairy and transitional foods.

**Table 1:** Food group factor loadings and mean intake (g) by PCA-derived dietary pattern tertile for Guatemalan women. INCAP Nutrition Supplementation Trial Longitudinal Cohort, 2002–2004 (*n* = 742)

	Meat-based modern				Starch-based modern				Traditional			
Food groups	Factor loading	T1^a^ (g)	T2 (g)	T3 (g)	Factor loading	T1 (g)	T2 (g)	T3 (g)	Factor loading	T1 (g)	T2 (g)	T3 (g)
Corn tortilla	0.23	343.9	397.7	390.4	0.03	389.2	382.6	359.3	0.70*	252.4	369.0	510.2
Refined grains	0.19	64.6	70.4	81.0	0.39*	46.2	73.3	96.8	0.01	70.2	70.4	75.3
Pastry	0.25	32.5	52.7	66.7	0.12	42.3	52.2	57.3	0.06	46.4	51.1	54.2
Salty snacks	0.49*	1.0	3.9	11.3	0.02	4.7	4.8	6.7	0.11	5.4	4.5	6.3
Non-starchy vegetables	0.08	90.0	77.9	93.0	0.52*	34.7	73.1	154.0	−0.14	116.6	78.8	65.3
Starchy vegetables	0.03	37.0	36.2	41.2	0.56*	12.5	29.1	73.3	−0.13	53.3	33.8	27.2
Fried starches	0.30	26.5	42.4	58.7	0.44*	18.4	35.6	73.7	−0.10	49.7	44.6	33.0
Fruits	0.07	225.6	196.0	231.4	0.39*	126.2	202.6	325.9	0.02	251.7	186.2	214.6
Eggs	0.35	19.4	29.8	37.6	0.21	22.3	29.5	34.9	0.31	20.1	30.9	35.8
Poultry	0.05	19.7	21.2	22.9	0.34	14.2	18.4	31.3	0.04	21.2	20.5	22.2
Red meat and pork	0.25	9.1	12.2	19.7	0.30	8.1	13.6	19.3	−0.24	18.7	13.0	9.3
Processed meats	0.39*	4.7	8.1	15.2	0.26	5.8	8.5	13.7	−0.05	10.8	8.8	8.3
Giblets	0.08	14.7	13.5	17.0	0.47*	5.3	10.4	29.7	0.07	12.4	15.8	17.1
Fish	0.23	5.6	9.6	13.0	0.21	4.3	6.7	17.3	0.14	9.1	7.7	11.3
Fried meats	0.51*	8.7	17.2	30.8	0.19	13.0	20.2	23.4	−0.09	22.5	17.6	16.5
Dairy	0.14	64.6	80.9	103.1	0.34	32.4	83.5	133.1	−0.36*	141.3	65.4	41.0
Beans	0.03	119.0	117.2	96.1	0.03	117.1	117.5	97.6	0.63	53.3	85.7	193.3
Oils/fats	0.24	6.6	9.8	13.7	0.37*	5.2	9.1	15.9	0.0	11.1	9.5	9.4
Sugar added to coffee	−0.06	12.3	13.6	12.6	−0.02	12.8	13.4	12.3	0.36*	7.5	11.6	19.4
Sweets	0.48*	1.2	3.1	8.6	0.13	3.5	3.4	5.9	−0.16	6.7	3.5	2.6
Alcohol	0.39*	0.1	0.3	5.7	−0.14	4.5	0.6	1.0	0.17	0.9	1.7	3.5
Sugar-sweetened beverages	0.01	307.4	297.6	300.7	0.45*	153.4	316.5	438.4	0.15	288.1	290.8	326.8
Low-energy drinks^b^	0.70*	24.1	70.1	206.3	−0.09	105.3	93.8	100.1	−0.06	113.0	100.7	85.6
Packaged soup	−0.06	17.3	12.9	12.3	0.37*	3.5	12.1	27.1	−0.08	18.7	11.1	12.7
Traditional Guatemalan foods^c^	0.36*	25.9	39.7	69.2	0.17	36.2	42.0	56.4	0.09	42.4	45.4	46.7
Transitional foods^d^	0.55*	1.0	1.4	8.4	0.07	3.2	2.5	5.2	−0.36*	8.0	1.9	0.8

Whole grains and nuts were excluded from analyses due to low (<10%) consumption

*Abbreviations*: *INCAP* Institute of Nutrition for Central America and Panama, *PCA* principal component analysis, *T* tertile

^a^Mean intake of food groups (g) across tertiles

^b^Sparkling/soda water and coconut water

^c^Tamales and tacos

^d^Pizza and hamburgers

^*^Food groups characterizing the dietary pattern (i.e. food groups with absolute factor loadings >0.35)

**Table 2:** Food group factor loadings and mean intake (g) by PCA-derived dietary pattern tertile for Guatemalan men. INCAP Nutrition Supplementation Trial Longitudinal Cohort, 2002–2004 (*n* = 681)

	Meat-based modern				Starch-based modern				Traditional			
Food groups	Factor loading	T1^a^ (g)	T2 (g)	T3 (g)	Factor loading	T1 (g)	T2 (g)	T3 (g)	Factor loading	T1 (g)	T2 (g)	T3 (g)
Corn tortilla	0.23	543.3	561.9	576.7	0.03	527.6	570.9	583.1	0.70*	406.3	547.0	728.5
Refined grains	0.19	81.0	89.9	104.8	0.39*	61.7	88.4	125.4	0.01	97.4	88.7	89.6
Pastry	0.25	55.5	59.7	69.6	0.12	54.7	65.1	64.9	0.06	60.6	59.3	64.8
Salty snacks	0.49*	3.0	9.4	19.2	0.02	11.8	9.8	9.9	0.11	8.7	10.9	11.9
Non-starchy vegetables	0.08	100.3	110.0	125.0	0.52*	56.4	92.0	186.7	−0.14	134.5	103.0	97.8
Starchy vegetables	0.03	40.0	41.3	44.0	0.56*	22.1	36.1	67.1	−0.13	46.1	43.3	36.0
Fried starches	0.30	34.6	50.7	71.9	0.44*	30.0	50.7	76.3	−0.10	62.3	50.4	44.4
Fruits	0.07	268.3	262.0	281.0	0.39*	146.9	267.4	396.4	0.02	264.7	239.8	306.7
Eggs	0.35	33.5	47.0	55.8	0.21	37.7	45.7	52.7	0.31	38.0	42.0	56.2
Poultry	0.05	19.4	19.9	22.3	0.34	12.7	19.7	29.3	0.04	19.0	19.3	23.4
Red meat and pork	0.25	11.0	16.2	21.4	0.30	11.3	13.3	23.9	−0.24	22.3	14.1	12.1
Processed meats	0.39*	7.0	15.0	25.5	0.26	9.9	15.7	21.9	−0.05	19.5	14.0	14.1
Giblets	0.08	12.6	15.2	19.7	0.47*	6.5	13.6	27.3	0.07	14.5	13.9	19.1
Fish	0.23	12.4	19.6		0.21	11.7	16.3	32.2	0.14	12.6	21.3	26.4
Fried meats	0.51*	12.8	24.8	40.5	0.19	21.3	24.8	31.9	−0.09	32.7	23.5	21.8
Dairy	0.14	49.2	66.3	91.3	0.34	35.2	64.2	107.0	−0.36*	99.6	56.2	50.8
Beans	0.03	137.0	154.3	139.0	0.03	134.6	143.9	151.8	0.63	78.7	111.4	240.3
Oils/fats	0.24	10.3	15.0	17.4	0.37*	8.3	13.3	21.1	0.0	14.7	14.3	13.8
Sugar added to coffee	−0.06	12.3	11.8	11.4	−0.02	11.9	11.7	11.8	0.36*	7.5	11.4	16.5
Sweets	0.48*	2.7	6.5	12.7	0.13	5.3	6.8	9.8	−0.16	9.9	6.0	5.9
Alcohol	0.39*	12.5	55.1	124.1	−0.14	100.1	45.4	46.1	0.17	42.9	61.5	87.0
Sugar-sweetened beverages	0.01	389.6	395.0	351.8	0.45*	194.8	377.5	563.4	0.15	329.5	368.8	438.3
Low-energy drinks^b^	0.70*	79.3	206.5	404.7	−0.09	283.8	218.1	187.9	−0.06	280.3	205.4	203.9
Packaged soup	−0.06	15.7	12.3	12.2	0.37*	6.8	9.9	23.6	−0.08	17.4	11.8	11.0
Guatemalan foods^c^	0.36*	29.2	48.1	79.6	0.17	41.9	53.7	61.2	0.09	44.0	56.3	56.4
Transitional foods^d^	0.55*	2.3	5.3	20.8	0.07	8.4	9.4	10.5	−0.36*	20.0	4.8	3.5

Whole grains and nuts were excluded from analyses due to low (<10%) consumption

*Abbreviations*: *INCAP* Institute of Nutrition for Central America and Panama, *PCA* principal component analysis, *T* tertile

^a^Mean intake of food groups (g) across tertiles

^b^Sparkling/soda water and coconut water

^c^Tamal and taco

^d^Pizza and hamburgers

^*^Food groups characterizing the dietary pattern (i.e. food groups with absolute factor loadings >0.35)

Higher meat-based modern pattern tertile was associated with lower SES in women (p trend 0.001) (Table 3) and younger age (p trend 0.002), and smoking (p trend 0.001) in men (Table 4). Higher starch-based modern pattern tertile was associated with multivitamin use in both sexes (p trend <0.001 for men and p trend 0.02 for women), and with physical inactivity (p trend 0.002) and higher BMI (p trend 0.02) in men. In both men and women, higher traditional pattern tertile was inversely associated with urban residence (p trend 0.008 and p trend <0.001, respectively). In men, higher traditional pattern tertile was also associated with lower SES (p trend 0.05), smoking (p trend 0.04), less physical inactivity (p trend <0.0001), lower BMI (p trend 0.003), less overweight (p trend 0.01), and less low HDL-c (p trend 0.009). Conversely, in women, higher traditional pattern tertile was associated with higher BMI (p trend 0.04).

**Table 3:** Sociodemographic characteristics, health outcomes, and nutrient intake by PCA-derived dietary pattern tertile for Guatemalan women. INCAP Nutrition Supplementation Trial Longitudinal Cohort, 2002–2004 (*n* = 747)

		Meat-based modern				Starch-based modern				Traditional			
	n	T1	T2	T3	*P*-trend^h^	T1	T2	T3	*P*-trend^h^	T1	T2	T3	*P*-trend^h^
Participants, n	747	252	247	248		252	247	248		251	246	250	
Mean factor score	747	−0.9 (0.2)	−0.5 (0.1)	0.3 (0.7)		−0.9 (0.2)	−0.2 (0.2)	1.0 (0.8)		−1.1 (0.4)	−0.3 (0.1)	0.6 (0.6)	
Age, y	747	33.4 (4.1)	32.6 (4.2)	32.8 (4.4)	0.1	32.7 (4.3)	32.7 (4.0)	33.3 (4.0)	0.1	32.0 (4.1)	33.0 (4.3)	32.7 (4.3)	0.5
SES tertile	747				0.001				0.6				0.6
Poorest		28.9	34.4	41.5		32.9	34.0	37.9		32.2	35.7	36.8	
Middle		23.8	29.7	22.9		27.7	25.9	20.9		27.4	24.8	22.4	
Richest		47.2	37.6	35.4		39.2	40.0	41.1		40.2	39.4	40.8	
Urban, %	747	28.5	29.9	29.8	0.7	25.4	30.7	32.2	0.09	44.6	26.4	17.2	<0.001
Current smoking, %	692	0.8	1.3	3.0	0.06^i^	2.1	2.1	0.8	0.2^i^	1.7	1.3	2.1	0.7^i^
Low physical activity^a^, %	747	97.2	97.9	95.5	0.2	98.0	95.1	97.5	0.7	96.4	98.3	96.0	0.7
Vitamin use, %	695	17.8	20.4	23.5	0.1	17.4	18.4	25.7	0.02	22.6	18.3	20.7	0.6
BMI (kg/m^2^)	683	26.8 (4.8)	26.8 (4.4)	26.9 (5.0)	0.6	27.1 (5.1)	26.9 (4.5)	26.7 (4.6)	0.4	26.4 (4.8)	27.0 (4.5)	27.3 (4.9)	0.04
Overweight^b^, %	683	59.3	61.7	65.6	0.1	60.7	63.4	62.3	0.7	58.5	64.2	63.7	0.2
Abdominal obesity^c^, %	683	61.5	63.0	62.0	0.9	63.0	61.3	62.3	0.8	55.9	66.5	64.2	0.06
Elevated triglycerides^d^, %	647	52.8	51.6	49.7	0.5	51.6	49.3	53.4	0.7	48.8	53.8	51.8	0.5
Low HDL-c^e^	647	82.6	87.2	87.2	0.1	84.8	85.3	86.6	0.5	82.4	86.5	87.8	0.1
Dysglycemia^f^	726	16.2	21.9	20.0	0.2	18.5	18.6	21.1	0.4	16.8	23.0	18.5	0.6
Metabolic Syndrome^g^	747	38.1	38.0	36.2	0.6	35.3	37.2	39.9	0.2	32.2	41.0	39.2	0.1
Dietary Intakes													
Total energy (kcal/day)	747	1864 (584)	2243 (582)	2646 (715)	<0.001	1813 (542)	2220 (559)	2722 (689)	<0.001	2010 (668)	2144 (591)	2593 (717)	<0.001
Carbohydrate (% of total energy)	747	71.7 (5.6)	68.9 (4.9)	65.8 (5.2)	<0.001	71.3 (5.3)	68.9 (5.2)	66.3 (5.7)	<0.001	66.1 (6.0)	69.2 (4.8)	71.3 (5.1)	<0.001
Protein (% of total energy)	747	12.1 (1.7)	12.1 (1.7)	12.4 (1.7)	0.05	12.0 (1.4)	12.2 (1.6)	12.6 (2.0)	<0.001	12.6 (2.1)	12.1 (1.5)	12.1 (1.3)	0.03
Fat (% of total energy)	747	16.0 (4.7)	18.7 (4.1)	21.6 (4.4)	<0.001	16.5 (4.9)	18.7 (4.3)	20.9 (4.6)	<0.001	21.2 (4.8)	18.6 (4.2)	16.4 (4.6)	<0.001
Cholesterol (mg/day)	747	194.3 (141.3)	274.6 (138.7)	367.8 (217.7)	<0.001	186.6 (135.8)	267.7 (159.0)	382.5 (196.5)	<0.001	246.4 (151.6)	284.0 (177.9)	305.2 (212.8)	0.002
Fiber (g/day)	747	25.3 (8.7)	28.3 (8.6)	30.8 (10.3)	<0.001	24.5 (8.0)	27.6 (8.8)	32.2 (10.0)	<0.001	23.5 (7.8)	26.8 (7.1)	33.9 (10.2)	<0.001
Iron (mg/day)	747	11.5 (5.3)	12.9 (4.5)	15.2 (5.3)	<0.001	9.5 (3.2)	12.7 (3.8)	17.4 (5.2)	<0.001	12.2 (4.9)	12.2 (4.9)	15.1 (5.5)	<0.001
Vitamin C		289.7 (217.5)	268.6 (186.4)	286.6 (183.7)	0.5	151.2 (105.6)	277.7 (146.3)	418.4 (218.8)	<0.001	291.9 (195.7)	258.2 (173.5)	294.5 (216.4)	0.7

Values presented are mean (SD) or percent

*Abbreviations*: *BMI* body mass index, *HDL-c* high density lipoprotein cholesterol, *INCAP* Institute of Nutrition for Central America and Panama, *NCEP ATP III* National Cholesterol Education Program Adult Treatment Panel III, *PCA* principal component analysis; SES, socioeconomic status, *T* tertile

^a^Low physical activity defined as 24-h average physical activity <1.7 MET/h

^b^Overweight defined as BMI ≥25 kg/m^2^

^c^Abdominal obesity defined as waist circumference ≥ 88 cm

^d^Elevated triglycerides defined as ≥150 mg/dL or medication

^e^Low HDL-c defined as HDL-c < 50 mg/dL

^f^Dysglycemia defined as impaired fasting glucose (fasting plasma glucose 100–125 mg/dL) or diabetes (self-report, fasting plasma glucose ≥126 mg/dL, and/or use of diabetes medication)

^g^Metabolic syndrome defined according to 2005 NCEP ATP III diagnostic criteria based on presence ≥3 of the following: abdominal obesity (waist circumference ≥ 88 cm); fasting plasma glucose ≥100 mg/dL or medication; triglycerides ≥150 mg/dL or medication; HDL-c < 50 mg/dL); blood pressure > 130 mmHg systolic, >85 mmHg diastolic and/or medication use

^h^P for trend was calculated using Spearman correlation (continuous) and Mantel-Haenszel Chi-square tests (categorical variables)

^i^Expected cell count <5

**Table 4:** Sociodemographic characteristics, health outcomes, and nutrient intake by PCA-derived dietary pattern tertile for Guatemalan men. INCAP Nutrition Supplementation Trial Longitudinal Cohort, 2002–2004 (*n* = 681)

		Meat-based modern				Starch-based modern				Traditional			
	n	T1	T2	T3	*P*-trend^h^	T1	T2	T3	*P*-trend^h^	T1	T2	T3	*P*-trend^h^
Participants, n	681	227	228	226		226	228	227		227	227	227	
Mean factor score	681	−0.5 (0.3)	0.2 (0.2)	1.6 (0.9)		−0.9 (0.4)	−0.06 (0.2)	1.2 (0.7)		−0.7 (0.6)	0.2 (0.2)	1.4 (0.6)	
Age, y	681	33.1 (4.2)	32.7 (4.3)	31.7 (3.9)	0.002	32.5 (4.3)	32.5 (4.1)	32.5 (4.2)	0.9	32.8 (4.4)	32.2 (4.1)	32.5 (4.0)	0.7
SES tertile	681				0.7				0.6				0.05
Poorest		36.5	31.1	33.6		32.7	30.2	38.3		28.6	37.0	35.6	
Middle		26.8	30.7	30.0		33.6	27.6	26.4		29.9	26.4	31.2	
Richest		36.5	38.1	36.2		33.6	42.1	35.2		41.4	36.5	33.0	
Urban, %	680	26.9	20.6	19.9	0.07	22.1	21.5	23.7	0.6	29.5	21.6	16.3	<0.001
Current smoking, %	554	32.1	43.8	48.3	0.001	46.8	36.6	40.4	0.2	36.2	41.0	46.5	0.04
Low physical activity^a^, %	681	61.2	56.5	53.5	0.09	49.1	58.7	63.4	0.002	66.9	56.8	47.5	<0.001
Vitamin use, %	554	24.2	21.6	26.0	0.6	15.9	24.0	32.2	<0.001	28.5	21.6	21.9	0.1
BMI (kg/m^2^)	589	24.5 (3.6)	24.5 (3.5)	24.7 (3.6)	0.5	24.3 (3.5)	24.5 (3.5)	25.0 (3.6)	0.02	25.0 (3.8)	24.8 (3.5)	24.1 (3.3)	0.003
Overweight^b^, %	589	38.9	41.6	42.3	0.4	38.2	37.5	47.1	0.07	46.7	42.6	34.1	0.01
Abdominal obesity^c^, %	589	4.9	6.6	6.3	0.5	6.1	4.5	7.2	0.6	7.0	6.0	4.8	0.3
Elevated triglycerides^d^, %	456	53.0	58.1	52.7	0.9	52.9	49.3	61.7	0.1	58.3	52.3	53.0	0.3
Low HDL-c^e^, %	456	74.3	76.3	72.2	0.6	68.3	78.2	76.5	0.1	82.6	70.2	69.8	0.009
Dysglycemia^f^, %	456	15.1	14.6	15.2	0.9	14.4	14.8	15.8	0.6	17.7	13.3	14.0	0.2
Metabolic syndrome^g^, %	681	14.5	15.7	14.6	0.9	15.9	14.4	14.5	0.6	18.0	12.7	14.1	0.2
Dietary Intakes													
Total energy (kcal/day)	681	2626 (676)	3059 (760)	3627 (839)	<0.001	2578 (733)	3064 (690)	3666 (796)	<0.001	2775 (862)	2945 (717)	3590 (784)	<0.001
Carbohydrate (% of total energy)	681	72.2 (4.5)	69.6 (4.4)	67.3 (5.4)	<0.001	71.2 (5.1)	70.0 (4.4)	67.9 (5.5)	<0.001	67.5 (5.1)	70.2 (4.9)	71.3 (4.9)	<0.001
Protein (% of total energy)	681	11.6 (1.6)	11.9 (1.4)	12.1 (1.6)	<0.001	11.6 (1.4)	11.8 (1.4)	12.3 (1.7)	<0.001	12.1 (1.6)	11.6 (1.5)	11.9 (1.5)	0.4
Fat (% of total energy)	681	16.0 (3.9)	18.4 (3.7)	20.5 (4.5)	<0.001	17.1 (4.6)	18.1 (3.8)	19.7 (4.5)	<0.001	20.2 (4.2)	18.0 (4.0)	16.6 (4.4)	<0.001
Cholesterol (mg/day)	681	280.9 (157.6)	396.8 (201.6)	501.6 (236.2)	<0.001	294.6 (174.3)	383.8 (195.6)	500.0 (236.0)	<0.001	365.9 (204.5)	361.5 (199.8)	451.3 (242.3)	<0.001
Fiber (g/day)	681	36.2 (11.6)	39.1 (13.0)	43.0 (13.1)	<0.001	33.5 (10.1)	39.3 (11.4)	45.4 (13.9)	<0.001	32.3 (10.6)	37.9 (9.8)	48.1 (12.7)	<0.001
Iron (mg/day)	681	14.6 (5.2)	17.1 (6.3)	19.8 (6.3)	<0.001	12.9 (4.7)	16.7 (4.3)	21.8 (6.3)	<0.001	15.7 (6.0)	15.7 (5.7)	20.0 (6.3)	<0.001
Vitamin C (mg/day)	681	352.5 (289.7)	362.2 (266.0)	352.0 (232.6)	0.6	191.2 (125.6)	351.2 (177.3)	523.7 (325.9)	<0.001	327.5 (212.1)	328.2 (215.0)	411.0 (336.2)	0.01

Values presented are mean (SD) or percent

*Abbreviations*: *BMI* body mass index, *HDL-c* high density lipoprotein cholesterol, *INCAP* Institute of Nutrition for Central America and Panama, *NCEP ATP III* National Cholesterol Education Program Adult Treatment Panel III, *PCA* principal component analysis, *SES* socioeconomic status, *T* tertile

^a^Low physical activity defined as 24-h average physical activity <1.7 MET/h

^b^Overweight defined as BMI ≥25 kg/m^2^

^c^Abdominal obesity defined as waist circumference ≥ 102 cm

^d^Elevated triglycerides defined as ≥150 mg/dL or medication

^e^Low HDL-c defined as HDL-c < 40 mg/dL

^f^Dysglycemia defined as impaired fasting glucose (fasting plasma glucose 100–125 mg/dL) or diabetes (self-report, fasting plasma glucose ≥126 mg/dL, and/or use of diabetes medication)

^g^Metabolic syndrome defined according to 2005 NCEP ATP III diagnostic criteria based on presence ≥3 of the following: abdominal obesity (waist circumference ≥ 88 cm); fasting plasma glucose ≥100 mg/dL or medication; triglycerides ≥150 mg/dL or medication; HDL-c < 50 mg/dL); blood pressure > 130 mmHg systolic, >85 mmHg diastolic and/or medication use

^h^P for trend was calculated using Spearman correlation (continuous) and Mantel- Haenszel Chi-square tests (categorical variables)

Within and across the three dietary patterns, carbohydrates provided the majority of energy intake ranging from 66 to 67% among participants in the highest tertile of the meat-based modern pattern to 72% among participants in the lowest tertile of this pattern (Tables 3 and 4). Participants in the lowest tertile of the traditional pattern had significantly lower fiber consumption (23.5 g for women and 32.3 g for men) compared to participants in the highest tertile (33.9 g for women and 48.1 g for men). Fat as a proportion of total energy intake ranged from 16.0% among men in the lowest tertile of the meat-based modern pattern to 21.6% among women in the highest tertile of the meat-based modern pattern. There were small but significant differences in protein consumption as a percentage of total energy intake (11.6% to 12.6%).

In women, relative to tertile 1, higher traditional diet pattern tertile was associated with increased prevalence of abdominal obesity (Prevalence Ratio (PR) 1.24, 95% Confidence Interval (CI) 1.07, 1.43 for tertile 2; PR 1.19, 95% CI 1.02, 1.39 for tertile 3; p trend 0.02) (Table 5). In men, relative to tertile 1, higher starch-based modern pattern tertile was associated with increased prevalence of low HDL-c (PR 1.17, 95% CI 1.01, 1.20 for tertile 2; PR 1.20, 95% CI 1.00, 1.44 for tertile 3; p trend 0.04) whereas higher traditional pattern tertile was marginally associated with reduced prevalence of low HDL-c (PR 0.88, 95% CI 0.76, 1.00 for tertile 2; PR 0.85, 95% CI 0.72, 1.00 for tertile 3; p trend 0.05) (Table 6). In men, higher traditional pattern tertile was also associated with decreased prevalence of overweight in models adjusting for age and total energy intake (Model 2 PR 0.94, 95% CI 0.75, 1.18 for tertile 2; PR 0.68, 95% CI 0.52, 0.88 for tertile 3; p trend 0.004) but there was no association after adjusting for physical inactivity, smoking, and multivitamin use in Model 3.

**Table 5:** Log binomial models predicting cardio-metabolic risk by PCA-derived dietary pattern tertile for Guatemalan women. INCAP Nutrition Supplementation Trial Longitudinal Cohort, 2002–2004. (*n* = 747)

	Meat-based modern			Starch-based modern			Traditional		
	T2 PR (95% CI)	T3 PR (95% CI)	P-trend	T2 PR (95% CI)	T3 PR (95% CI)	P-trend	T2 PR (95% CI)	T3 PR (95% CI)	P-trend
Overweight^a^									
Model 1	1.05 (0.90, 1.21)	1.10 (0,96, 1.27)	0.1	1.03 (0.89, 1.19)	1.00 (0.87, 1.16)	0.8	1.09 (0.94, 1.26)	1.07 (0.92, 1.23)	0.3
Model 2	1.03 (0.88, 1.20)	1.07 (0.92, 1.25)	0.3	0.98 (0.84, 1.14)	0.92 (0.77, 1.10)	0.3	1.08 (0.93, 1.25)	1.04 (0.89, 1.21)	0.5
Model 3	1.03 (0.89, 1.20)	1.08 (0.92, 1.25)	0.3	0.97 (0.84, 1.12)	0.89 (0.76, 1.05)	0.1	1.11 (0.96, 1.28)	1.11 (0.96, 1.30)	0.1
Abdominal obesity^b^									
Model 1	1.02 (0.88, 1.17)	0.97 (0.84, 1.12)	0.9	0.96 (0.83, 1.10)	0.97 (0.84, 1.12)	0.7	1.18 (1.02, 1.37)	1.14 (0.98, 1.32)	0.07
Model 2	0.99 (0.85, 1.14)	0.95 (0.81, 1.12)	0.5	0.91 (0.78, 1.06)	0.88 (0.74, 1.05)	0.1	1.18 (1.07, 1.37)	1.11 (0.95, 1.30)	0.1
Model 3	1.00 (0.87, 1.16)	0.97 (0.83, 1.13)	0.7	0.90 (0.78, 1.05)	0.85 (0.72, 1.00)	0.06	1.24 (1.07, 1.43)	1.19 (1.02, 1.39)	0.02
Elevated triglycerides^c^									
Model 1	0.97 (0.81, 1.17)	0.94 (0.78, 1.13)	0.5	0.97 (0.80, 1.17)	1.04 (0.86, 1.25)	0.6	1.09 (0.91, 1.32)	1.04 (0.86, 1.25)	0.1
Model 2	0.96 (0.80, 1.16)	0.92 (0.75, 1.13)	0.4	0.97 (0.80, 1.19)	1.05 (0.85, 1.30)	0.6	1.09 (0.91, 1.32)	1.04 (0.85, 1.27)	0.6
Model 3	0.98 (0.81, 1.19)	0.94 (0.76, 1.15)	0.5	0.99 (0.81, 1.20)	1.08 (0.87, 1.34)	0.4	1.06 (0.87, 1.19)	1.01 (0.82, 1.24)	0.8
Low HDL-c^d^									
Model 1	1.05 (0.99, 1.12)	0.99 (0.90, 1.90)	0.8	0.99 (0.92, 1.07)	1.02 (0.94, 1.10)	0.6	1.02 (0.94, 1.11)	1.05 (0.98, 1.14)	0.1
Model 2	1.08 (1.00, 1.17)	1.08 (0.99, 1.19)	0.07	1.00 (0.92, 1.08)	1.03 (0.94, 1.13)	0.4	1.03 (0.95, 1.11)	1.06 (0.98, 1.14)	0.1
Model 3	1.09 (1.01, 1.18)	1.09 (0.99, 1.20)	0.06	1.00 (0.92, 1.08)	1.03 (0.93, 1.14)	0.5	1.04 (0.94, 1.11)	1.06 (0.98, 1.15)	0.1
Dysglycemia^e^									
Model 1	1.46 (1.00, 2.12)	1.28 (0.86, 1.89)	0.2	1.01 (0.69, 1.47)	1.07 (0.74, 1.54)	0.7	1.28 (0.89, 1.80)	1.03 (0.70, 1.51)	0.8
Model 2	1.48 (1.00, 2.17)	1.30 (0.85, 2.00)	0.2	1.00 (0.68, 1.47)	1.04 (0.68, 1.60)	0.8	1.27 (0.88, 1.83)	1.00 (0.66, 1.50)	0.9
Model 3	1.46 (0.99, 2.14)	1.26 (0.82, 1.94)	0.2	0.99 (0.67, 1.45)	1.04 (0.68, 1.59)	0.8	1.29 (0.89, 1.87)	1.03 (0.68, 1.56)	0.8
Metabolic syndrome^f^									
Model 1	1.00 (0.80, 1.24)	0.97 (0.78, 1.21)	0.8	1.01 (0.81, 1.27)	1.08 (0.87, 1.34)	0.4	1.23 (0.98, 1.55)	1.15 (0.91, 1.45)	0.2
Model 2	0.98 (0.78, 1.22)	0.94 (0.73, 1.20)	0.6	1.01 (0.80, 1.28)	1.07 (0.83, 1.38)	0.5	1.23 (0.98, 1.54)	1.14 (0.90, 1.46)	0.2
Model 3	1.00 (0.80, 1.25)	0.95 (0.74, 1.21)	0.6	1.00 (0.79, 1.26)	1.04 (0.81, 1.35)	0.7	1.27 (1.01, 1.59)	1.18 (0.93, 1.51)	0.1

*Abbreviations*: *BMI* body mass index, *CI* confidence interval, *HDL-c* high density lipoprotein cholesterol, *INCAP* Institute of Nutrition for Central America and Panama, *NCEP ATP III* National Cholesterol Education Program Adult Treatment Panel III, *PCA* principal component analysis, *PR* prevalence ratio, *SES* socioeconomic status, *T* tertile

Model 1 = dietary pattern tertile + age

Model 2 = model 1 + kcal/day

Model 3 = model 2 + SES + urban residence + multivitamin use

^a^Overweight defined as BMI ≥25 kg/m^2^

^b^Abdominal obesity defined as waist circumference ≥ 88 cm

^c^Elevated triglycerides defined as ≥150 mg/dL or medication

^d^Low HDL-c defined as HDL-c < 50 mg/dL

^e^Dysglycemia defined as impaired fasting glucose (fasting plasma glucose 100–125 mg/dL) or diabetes (self-report, fasting plasma glucose ≥126 mg/dL, and/or use of diabetes medication)

^f^Metabolic syndrome defined according to 2005 NCEP ATP III diagnostic criteria based on presence ≥3 of the following: abdominal obesity (waist circumference ≥ 88 cm); fasting plasma glucose ≥100 mg/dL or medication; triglycerides ≥150 mg/dL or medication; HDL-c < 50 mg/dL); blood pressure > 130 mmHg systolic, >85 mmHg diastolic and/or medication use

**Table 6:** Log binomial models predicting cardio-metabolic risk by PCA-derived dietary pattern tertile for Guatemalan men. INCAP Nutrition Supplementation Trial Longitudinal Cohort, 2002–2004. (*n* = 681)

	Meat-based modern			Starch-based modern			Traditional		
	T2 PR (95% CI)	T3 PR (95% CI)	P-trend	T2 PR (95% CI)	T3 PR (95% CI)	P-trend	T2 PR (95% CI)	T3 PR (95% CI)	P-trend
Overweight^a^									
Model 1	1.07 (0.84, 1.37)	1.15 (0.90, 1.47)	0.2	1.03 (0.80, 1.32)	1.24 (0.98, 1,57)	0.06	0.96 (0.76, 1.20)	0.78 (0.61, 0.99)	0.04
Model 2	1,05 (0.82, 1.34)	1.07 (0.82, 1.41)	0.5	1.01 (0.78, 1.31)	1.18 (0.89, 1.56)	0.2	0.94 (0.75, 1.18)	0.68 (0.52, 0.88)	0.004
Model 3	1.08 (0.87, 1.33)	1.15 (0.91, 1.45)	0.9	0.91 (0.72, 1.16)	0.98 (0.75, 1.27)	0.9	0.98 (0.80, 1.19)	0.84 (0.67, 1.05)	0.9
Abdominal obesity^b^									
Model 1	1.35 (0.56, 3.24)	1.53 (0.63, 3.71)	0.6	0.74 (0.30, 1.81)	1.15 (0.50, 2.62)	0.6	0.94 (0.41, 2.16)	0.64 (0.26, 1.54)	0.5
Model 2	1.25 (0.55, 2.84)	1.26 (0.49, 3.24)	0.6	0.68 (0.29, 1.61)	0.92 (0.39, 2.17)	0.8	0.95 (0.76, 1.20)	1.13 (0.90, 1.42)	0.2
Model 3	1.36 (0.53, 3.45)	1.75 (0.60, 5.15)	0.5	0.58 (0.22, 1.54)	0.56 (0.19, 1.61)	0.4	1.19 (0.49, 2.84)	0.61 (0.21, 1.73)	0.4
Elevated triglycerides^c^									
Model 1	1.11 (0.91, 1.36)	1.03 (0.83, 1.29)	0.7	0.96 (0.77, 1.20)	1.16 (0.95, 1.42)	0.1	0.91 (0.74, 1.11)	0.92 (0.75, 1.,13)	0.4
Model 2	1.10 (0.89, 1.34)	0.97 (0.76, 1.23)	0.8	0.95 (0.76, 1.20)	1.13 (0.90, 1.42)	0.1	0.89 (0.72, 1.09)	0.85 (0.68, 1.06)	0.1
Model 3	1.12 (0.91, 1.37)	1.03 (0.81, 1.31)	0.7	0.92 (0.73, 1.16)	1.05 (0.83, 1.33)	0.6	0.88 (0.74, 1.11)	0.79 (0.62, 1.01)	0.06
Low HDL-c^d^									
Model 1	1.02 (0.89, 1.16)	0.96 (0.83, 1.11)	0.6	1.15 (1.00, 1.32)	1.11 (0.96, 1.28)	0.1	0.86 (0.76, 0.98)	0.85 (0.74, 0.97)	0.01
Model 2	1.03 (0.90, 1.17)	0.99 (0.84, 1.17)	0.9	1.18 (1.03, 1.37)	1.21 (1.01, 1.44)	0.03	0.85 (0.74, 0.98)	0.83 (0.71, 0.97)	0.02
Model 3	1.07 (0.94, 1.23)	1.02 (0.86, 1.20)	0.7	1.17 (1.01, 1.35)	1.20 (1.00, 1.44)	0.04	0.88 (0.76, 1.00)	0.85 (0.72, 1.00)	0.05
Dysglycemia^e^									
Model 1	0.93 (0.57, 1.49)	1.08 (0.68, 1.72)	0.7	0.95 (0.59, 1.54)	1.10 (0.69, 1.75)	0.6	0.84 (0.52, 1.34)	0.84 (0.53, 1.33)	0.4
Model 2									
Model 3	0.98 (0.60, 1.57)	1.19 (0.70, 2.02)	0.5	1.03 (0.62, 1.70)	1.13 (0.65, 1.98)	0.6	0.83 (0.52, 1.33)	0.79 (0.47, 1.33)	0.3
Metabolic syndrome^f^									
Model 1	1.21 (0.78, 1.88)	1.17 (0.74, 1.84)	0.4	0.88 (0.56, 1.36)	0.94 (0.61, 1.46)	0.8	0.77 (0.49, 1.19)	0.76 (0.49, 1.17)	0.2
Model 2	0.94 (0.58, 1.53)	1.14 (0.67, 1.93)	0.6	0.98 (0.60, 1.61)	1.17 (0.69, 1.98)	0.5	0.84 (0.52, 1.34)	0.83 (0.50, 1.38)	0.4
Model 3	1.35 (0.88, 2.06)	1.48 (0.89, 2,.46)	0.1	0.89 (0.56, 1.41)	0.88 (0.53, 1.47)	0.6	0.79 (0.51, 1.21)	0.82 (0.51, 1.32)	0.4

*Abbreviations*: *BMI* body mass index, *CI* confidence interval, *HDL-c* high density lipoprotein cholesterol, *INCAP* Institute of Nutrition for Central America and Panama, *NCEP ATP III* National Cholesterol Education Program Adult Treatment Panel III, *PCA* principal component analysis, *PR* prevalence ratio, *SES* socioeconomic status, *T* tertile

Model 1 = dietary pattern tertile + age

Model 2 = model 1 + kcal/day

Model 3 = model 2 + SES + urban residence + multivitamin use + smoking status (current vs. other) + low physical activity physical activity

^a^Overweight defined as BMI ≥25 kg/m^2^

^b^Abdominal obesity defined as waist circumference ≥ 102 cm

^c^Elevated triglycerides defined as ≥150 mg/dL or medication

^d^Low HDL-c defined as HDL-c < 40 mg/dL

^e^Dysglycemia defined as impaired fasting glucose (fasting plasma glucose 100–125 mg/dL) or diabetes (self-report, fasting plasma glucose ≥126 mg/dL, and/or use of diabetes medication)

^f^Metabolic syndrome defined according to 2005 NCEP ATP III diagnostic criteria based on presence ≥3 of the following: abdominal obesity (waist circumference ≥ 88 cm); fasting plasma glucose ≥100 mg/dL or medication; triglycerides ≥150 mg/dL or medication; HDL-c < 50 mg/dL); blood pressure > 130 mmHg systolic, >85 mmHg diastolic and/or medication use

We did not find evidence of effect modification between dietary pattern tertile and urban residence on any CMD risk factors (*P* > 0.1 for all comparisons).

## Discussion

We identified three dietary patterns in this population: a traditional pattern and two modern patterns (meat-based and starch-based), collectively explaining one-quarter of variation in the diet. Variation explained is slightly higher in our study relative to dietary pattern analyses in other Latin American populations where percent variance explained was reported (20.4% in a population of urban Mexican adults) [36]. All three dietary patterns were characterized by high carbohydrate consumption (66-72% of total energy intake) with corn tortillas being the principal contributor, even among participants with high modern diet pattern scores. The traditional diet was associated with rural residence in both sexes and with lower SES, smoking, and physical activity in men. In fully adjusted models, higher traditional diet tertile was associated with increased prevalence of abdominal obesity in women but marginally reduced prevalence of low HDL-c in men. Higher starch-based modern diet tertile was associated with increased prevalence of low HDL-c in men.

The nutrition transition is characterized by a shift from traditional diets comprised of whole foods including pulses and whole grains to a modern diet comprised of refined carbohydrates, high fat, and processed foods [3, 37, 38]. In this Guatemalan population, we found evidence of two dominant modern dietary patterns – one related to energy-dense, nutrient-poor foods (meat-based) and a second with a mixture of processed less healthy foods and more healthy foods (starch-based). The meat-based modern pattern was characterized by consumption of salty snacks, processed and fried meats, soda, alcohol, candies, and transitional foods. While the meat-based modern pattern typifies the unhealthy modern diet associated with the nutrition transition, shifts to modern diets do not necessarily exclude additions of healthy foods. The starch-based modern pattern was characterized by both less healthy modern foods including refined grains and fried starches but also healthy foods such as non-starchy vegetables and fruits, which are not typically consumed in large quantities in the traditional Guatemalan diet. Thus, the starch-based modern pattern is an example of a transitional diet that is not wholly unhealthy. Studies from China found improved dietary diversity with the nutrition transition through greater inclusion of fruits and vegetables, eggs, dairy, and meat [3] while data from the Food and Agriculture Organization national food balance sheets in low- and middle-income countries (LMICs) show increased availability of processed foods and beverages [39].

The traditional pattern appeared to be associated with reduced CMD prevalence in men for low HDL-c but increased prevalence in women for abdominal obesity. Associations between traditional pattern in men and reduced prevalence of obesity were attenuated after controlling for lifestyle factors. Other studies in Latin American populations have not yielded consistent findings concerning traditional diets and CMD risk - possibly due to differences in fruit and vegetable consumption. The traditional rice and beans dietary pattern was associated with higher risk of metabolic syndrome and lower HDL-c in a cohort of Puerto Rican adults residing in Massachusetts [40]. Among women of Mexican descent living in the U.S., the traditional Mexican diet was associated with lower C-reactive protein and insulin concentrations [22]. Comparing these studies is complicated because the concept of “traditional” and “modern” diets varies across cultures and regions. The traditional rice and beans pattern in the study of Puerto Rican adults was characterized by oils, rice, beans/legumes, and vegetables [40]. The traditional Mexican diet was characterized by high intake of fresh fruits and vegetables, whole grains, and legumes and low intakes of refined carbohydrates and sugars [22]. Balanced diets high in fruits and vegetables, whole grains, and healthy fats have been associated with reduced obesity and diabetes [41, 42]. However, the traditional diet in Guatemala is characterized by consumption of corn tortillas, beans, and sweetened coffee with relatively low intake of vegetables, fruit, and healthy fats. Thus, the traditional Guatemalan diet would not necessarily be expected to be associated with reduced CMD risk.

The study population’s relatively homogenous diet with high reliance on carbohydrates (66-72% of total energy intake) could explain why we detected few associations between dietary patterns and CMD risk. Corn tortillas are the primary source of carbohydrates and constitute a large proportion of the diet - even among participants with high modern diet pattern scores. Corn tortillas are a good source of fiber (approximately 6 g per 2 oz. serving) – likely explaining adequate fiber intakes for most participants relative to U.S. Dietary Reference Intakes (25 g for women and 38 g for men) [43]. While fiber is associated with reduced cardiovascular risk [44], NHLBI recommends consuming <60% of calories from carbohydrates to help prevent dyslipidemia while the Institutes of Medicine recommends no more than 65% [30, 43]. Minor differences in the macronutrient composition of the traditional and modern diet patterns were due to small substitutions of corn tortillas for wheat-based breads, crackers, and other refined carbohydrates. This “modernization” of the diet has been seen elsewhere in Guatemala [45, 46]. Because of the way in which they are metabolized, corn tortillas and refined grains have similar effects on blood glucose and triglycerides [47], potentially diminishing differences in CMD risk by dietary pattern.

The absolute intake of modern foods in this population was relatively low – even in the highest tertiles of the two modern diet patterns. For example, men and women in the highest tertile of the meat-based modern diet pattern consumed an average of 19 and 11 g of salty snack foods per day, respectively, or less than a typical single-serving bag of potato chips (about 28 g). However, there was high added sugar consumption. Sweets and powdered drink/sugar-sweetened beverage consumption accounted for approximately 17% of energy intake – nearly double the 10% limit for free sugars recommended by the World Health Organization to prevent chronic disease [18, 48]. While not a “modern” food, coffee, through its association with added sugars, could be an important driver of poor nutrition. In the highest tertile of the traditional pattern, women and men added an average of 4.8 tsp. and 4.1 tsp. of sugar, respectively, to their coffee. Sugar-sweetened beverages are positively associated with body weight [49], and added sugars from liquid sources are associated with higher fasting glucose, higher fasting insulin, and higher β-cell dysfunction and insulin resistance [50].

Sociodemographic and health behaviors tracked as expected with the dietary patterns; however, a few observations are worth highlighting here. First, both modern dietary patterns were associated with unhealthy behaviors (alcohol consumption and smoking with the meat-based modern pattern and physical inactivity with the starch-based modern pattern) in men but not women; however, lack of variation in smoking and physical inactivity in women limited our ability to detect any trends. Second, modern dietary patterns are thought to track with income in LMICs where higher income households benefit from increased access to market foods [51]. In Guatemala, higher household annual expenditures were associated with increased likelihood of consuming a Western diet [9] and higher SES was associated with increased household-level calorie and processed food intake [52]. Conversely, we found that the meat-based modern pattern was associated with lower SES among women. These women might be purchasing cheap, processed foods rather than items typically associated with higher income such as dairy and unprocessed meats. Finally, at the beginning of the nutrition transition, dietary changes first appear in urban populations [2], likely due to differences in infrastructure, employment, income, and food access [51]. We found that while the traditional pattern was more dominant in rural areas, neither of the modern diet patterns varied by urban residence, suggesting that while urban residents are less likely to eat a traditional diet, there is considerable variation in dietary patterns among urban dwellers.

There are several possible explanations for the apparent sex differences in the different physiological responses to dietary components including sex-specific nutrient-gene interactions [53], the influence of sex hormones on metabolic risk factors (triglycerides and HDL-c) [54], or differences in dietary intake not captured by our patterns. One limitation of dietary pattern analysis is the inability to detect the biologic effects of specific nutrients [49], thus our study cannot draw conclusions about the biologic mechanisms responsible for the sex differences. However, studies from European and Korean populations have shown that dietary patterns might influence CMD risk (BMI, metabolic syndrome, metabolic syndrome components) differently in men and women [55–57].

### Strengths and limitations

There are some limitations to the dietary data. FFQs can obscure food choice-differences within food groups; for example, low- versus high-fat dairy [13]. We also may not have fully captured processed food consumption; however, the FFQ was designed to capture consumption of “transitional” foods, sweets, and sugar-sweetened beverages typically associated with modern diets. As Guatemala continues to develop, the rural/urban dichotomy is likely more nuanced than we present here. Nevertheless, because urban residence was based on residence location, neighborhood, and amenities, we expect it captures more than simple census-based classifications.

This study also has a number of strengths. The FFQ was developed for and validated in this population [21]. It had open-ended sections for fruit and vegetable consumption to capture potential seasonality in the diet and included a number of “transitional” foods relevant to identifying modern diets. The study used clinically measured anthropometry and biomarkers of CMD risk. The dietary patterns were relatively stable when the study sample was randomly reduced from 1428 to 717 participants in terms of percent variance explained (25.7%, collectively) and similarity of food groups and food group factor loadings (Additional file 2: Table S2). Even though the population is not nationally or provincially representative, results contribute to the very limited literature on dietary patterns in a Latin American country. Few studies report dietary patterns outside of high-income countries, yet populations in LMICs likely have different dietary patterns [58–62]. To our knowledge, this is the first paper to explore the association between PCA-derived dietary patterns and CMD risk in Central American adults.

## Conclusion

Our findings suggest the emergence of two modern diet patterns in Guatemala – one of which was associated with increased prevalence of low HDL-c in men. The traditional diet appeared to have a differential association with some CMD risk factors by sex. All three dietary patterns were characterized by carbohydrate consumption in excess of recommended levels – possibly explaining why we detected few associations between diet pattern and CMD risk factors. Future research will explore longitudinal diet and CMD risk in this population and quantify the contribution of other risk factors relative to diet in predicting CMD risk.

## Additional files

Additional file 1: Table S1. Food groups and food items from the food frequency questionnaire (52 food items plus 62 free-listed fruits and vegetables). INCAP Nutrition Supplementation Trial Longitudinal Cohort, 2002–2004. (DOCX 29 kb)

Additional file 2: Table S2. Food group factor loadings and mean intake (g) by PCA-derived dietary pattern tertile for a randomly split study sample of Guatemalan adults to assess internal validity of dietary patterns. INCAP Nutrition Supplementation Trial Longitudinal Cohort, 2002–2004 (*n* = 373 women, *n* = 344 men). (DOCX 37 kb)

## Abbreviations

BMI: Body mass index
CI: Confidence interval
CMD: Cardio-metabolic disease
FFQ: Food frequency questionnaire
HDL-c: High density lipoprotein cholesterol
INCAP: Institute of Nutrition of Central America and Panama
LMICs: Low- and middle-income countries
NCD: Non-communicable disease
NCEP ATP III: National Cholesterol Education Program Adult Treatment Panel III
PAL: Physical activity level
PCA: Principal components analysis
PR: Prevalence ratio
SES: Socioeconomic status
