# Supplementary material for: Dietary patterns and cardio-metabolic risk in a population of Guatemalan young adults
Source: BMC Nutr. 2017 Jul 28;3:68. doi: 10.1186/s40795-017-0188-5 (PMC5993443; doi:10.1186/s40795-017-0188-5)
Supplement: Supplementary file 1 — Food groups and food items from the food frequency questionnaire (52 food items plus 62 free-listed fruits and vegetables). INCAP Nutrition Supplementation Trial Longitudinal Cohort, 2002–2004. (DOCX 29 kb) [file 40795_2017_188_MOESM1_ESM.docx]

| Table S1. Food groups and food items from the food frequency questionnaire (52 food items plus 62 free-listed fruits and vegetables). INCAP Nutrition Supplementation Trial Longitudinal Cohort, 2002-2004. | | |
| --- | --- | --- |
| Main group | Food groups | Food items |
| Grains | Corn tortilla | Corn tortilla |
|  | Whole grains | Oatmeal |
|  | Refined grains | White bread, rice (boiled), rice (fried), corn flakes, noodles |
|  | Pastry | Sweet breads, pastries |
|  | Salty snacks | Chips (Papalinas/tortrix/ricitos) |
|  |  |  |
| Vegetables | Non-starchy vegetables | Carrot, herbs, squash (guicoy sazon)  Free listed non-starchy vegetables: avocado, beet, broccoli, cabbage, cactus (nopal), cauliflower, celery, chayote, cucumber, eggplant, greens, green beans, lettuce, loroco flower, okra, onion, pacaya palm, radish, soy, sweet pepper, squash (guicoy tierno), squash (other), tomato, yucca flower |
|  | Tubers/starchy vegetables | Potato (boiled), plantain (boiled)  Free listed tubers/starchy vegetables: peas, corn, chayote root, turnip, |
|  | Fried starches | French fries, fried plantains |
|  |  |  |
| Fruits | Fresh fruits | Free listed fruits: apple, banana (various types), blackberry, cashew fruit, chicozapote, coconut, grapes, green mango, grapefruit, guanabana, guava, jocote (green), jocote (yellow), jocote (red), leechee, mandarin orange, mamey, mango, melon, nance, orange, papaya, passion fruit, paterna, peach, pear, pineapple, plum, strawberry, sweet lime, sugar apple, sunza, watermelon, zapote |
|  |  |  |
| Meats | Eggs | Eggs |
|  | Poultry | Chicken (breast, leg, thigh, wing) |
|  | Red meat and pork | Beef/pork |
|  | Processed meat | Pork sausage, hot dog, ham |
|  | Giblets | Chicken giblets (liver, gizzard, feet), beef giblets (brain, kidney, liver, other) |
|  | Fish | Fish |
|  | Fried meat | Chicken (wing, thigh, leg, breast), pork skin |
|  |  |  |
| Dairy | Dairy | Milk, cheese |
|  |  |  |
| Beans | Beans | Beans (boiled), beans (refried/smashed) |
|  |  |  |
| Fats | Nuts | Peanuts |
|  | Oils/fats | Cream, butter/margarine, mayonnaise |
|  |  |  |
| Sugar | Sugar | Sugar added to coffee |
|  | Sweets | Candies/caramels/ chocolates, ice cream, jelly |
|  |  |  |
| Beverages | Coffee | Coffee |
|  | Alcohol | Beer, rum/whisky/other liquor |
|  | Sugar-sweetened beverages | Soft drinks, lemonade |
|  | Low-energy drinks | Coconut water, soda water |
|  |  |  |
| Other | Processed soup | Dried/packaged soup mix |
|  | Guatemalan foods | Tamale, taco/tostada |
|  | Transitional foods | Hamburger, pizza |
| Abbreviations: INCAP, Institute of Nutrition for Central America and Panama. | | |
