# Supplementary material for: Dietary patterns and cardio-metabolic risk in a population of Guatemalan young adults
Source: BMC Nutr. 2017 Jul 28;3:68. doi: 10.1186/s40795-017-0188-5 (PMC5993443; doi:10.1186/s40795-017-0188-5)
Supplement: Supplementary file 2 — Food group factor loadings and mean intake (g) by PCA-derived dietary pattern tertile for a randomly split study sample of Guatemalan adults to assess internal validity of dietary patterns. INCAP Nutrition Supplementation Trial Longitudinal Cohort, 2002–2004 (n = 373 women, n = 344 men). (DOCX 37 kb) [file 40795_2017_188_MOESM2_ESM.docx]

| Table S2. Food group factor loadings and mean intake (g) by PCA-derived dietary pattern tertile for a randomly split study sample of Guatemalan adults to assess internal validity of dietary patterns. INCAP Nutrition Supplementation Trial Longitudinal Cohort, 2002-2004 (n=373 women, n=344 men). | | | | | | | | | | | | |
| --- | --- | --- | --- | --- | --- | --- | --- | --- | --- | --- | --- | --- |
|  | Meat-based Modern | | | | Starch-based Modern | | | | Traditional | | | |
| Food Groups | Factor loading | T1^1^  (g) | T2  (g) | T3  (g) | Factor loading | T1  (g) | T2  (g) | T3  (g) | Factor loading | T1  (g) | T2  (g) | T3  (g) |
| WOMEN | | | | | | | | | | | | |
| Corn tortilla | 0.26 | 354.2 | 404.0 | 390.7 | 0.03 | 397.9 | 379.0 | 370.2 | 0.65* | 261.4 | 385.0 | 507.7 |
| Refined grains | 0.09 | 68.4 | 75.2 | 77.0 | 0.40* | 47.9 | 73.4 | 99.2 | 0.03 | 72.9 | 69.1 | 79.0 |
| Pastry | 0.14 | 42.2 | 50.6 | 52.9 | 0.26 | 35.8 | 48.2 | 61.5 | 0.21 | 40.4 | 43.6 | 62.4 |
| Salty snacks | 0.57* | 0.78 | 1.8 | 13.7 | 0.07 | 3.2 | 6.0 | 7.4 | 0.06 | 5.9 | 4.7 | 6.1 |
| Non-starchy vegetables | 0.13 | 77.0 | 85.6 | 93.7 | 0.48* | 39.5 | 74.7 | 142.1 | -0.15 | 124.4 | 72.5 | 58.4 |
| Starchy vegetables | 0.05 | 37.5 | 34.4 | 50.3 | 0.48* | 13.7 | 28.7 | 80.4 | -0.14 | 59.5 | 32.1 | 31.0 |
| Fried starches | 0.25 | 24.8 | 43.3 | 58.7 | 0.43* | 20.1 | 32.0 | 74.8 | -0.11 | 53.2 | 40.4 | 32.7 |
| Fruits | -0.03 | 250.3 | 172.1 | 217.6 | 0.48* | 110.3 | 206.9 | 326.5 | 0.03 | 252.1 | 164.5 | 229.8 |
| Eggs | 0.31 | 18.7 | 28.5 | 33.6 | 0.25 | 20.8 | 26.9 | 33.0 | 0.38* | 16.7 | 31.1 | 33.1 |
| Poultry | -0.04 | 20.4 | 17.6 | 23.3 | 0.38* | 14.2 | 17.0 | 30.4 | 0.10 | 21.6 | 18.3 | 21.9 |
| Red meat and pork | 0.20 | 7.6 | 13.4 | 19.4 | 0.33 | 7.0 | 14.8 | 18.6 | -0.29 | 21.0 | 11.1 | 8.2 |
| Processed meats | 0.15 | 7.6 | 8.2 | 12.1 | 0.42* | 4.3 | 8.4 | 15.4 | 0.07 | 7.8 | 9.9 | 10.3 |
| Giblets | 0.14 | 10.5 | 12.4 | 22.4 | 0.37* | 6.4 | 12.0 | 27.1 | -0.03 | 18.2 | 13.0 | 14.3 |
| Fish | 0.29 | 4.2 | 6.8 | 17.8 | 0.05 | 5.7 | 8.5 | 14.9 | -0.06 | 14.5 | 9.1 | 5.2 |
| Fried meats | 0.49* | 9.5 | 16.6 | 28.1 | 0.26 | 15.0 | 17.6 | 21.7 | 0.04 | 17.7 | 17.4 | 19.3 |
| Dairy | 0.06 | 70.8 | 80.1 | 87.8 | 0.39* | 27.0 | 84.9 | 126.7 | -0.29 | 132.4 | 62.1 | 42.7 |
| Beans | -0.09 | 141.8 | 107.2 | 84.7 | 0.05 | 111.6 | 119.3 | 103.2 | 0.65* | 50.7 | 87.7 | 201.8 |
| Oils/fats | 0.18 | 8.1 | 8.9 | 12.3 | 0.43* | 4.8 | 8.6 | 16.1 | 0.04 | 11.4 | 8.8 | 9.1 |
| Sugar added to coffee | -0.07 | 13.8 | 13.1 | 11.6 | -0.05 | 13.3 | 12.9 | 12.2 | 0.39* | 7.1 | 10.3 | 21.6 |
| Sweets | 0.51* | 1.2 | 2.9 | 10.4 | 0.23 | 2.9 | 3.5 | 8.3 | -0.19 | 8.4 | 3.0 | 3.2 |
| Alcohol | 0.45* | 0.18 | 0.17 | 9.9 | -0.19 | 9.6 | 0.09 | 0.89 | 0.09 | 0.89 | 3.6 | 6.1 |
| Sugar-sweetened beverages | 0.11 | 271.4 | 276.1 | 339.5 | 0.39* | 178.8 | 314.6 | 395.1 | 0.21 | 262.5 | 288.1 | 340.9 |
| Low-energy drinks^2^ | 0.68* | 6.2 | 76.7 | 234.2 | 0.04 | 102.7 | 108.1 | 129.4 | -0.02 | 129.5 | 112.5 | 97.3 |
| Packaged soup | -0.21 | 22.3 | 8.8 | 11.3 | 0.39* | 2.7 | 12.1 | 28.1 | -0.02 | 13.3 | 16.3 | 13.1 |
| Traditional Guatemalan foods^3^ | 0.37* | 26.7 | 36.4 | 72.6 | 0.18 | 36.2 | 46.8 | 53.5 | 0.14 | 43.3 | 45.1 | 48.3 |
| Transitional foods^4^ | 0.39* | 1.3 | 2.8 | 7.9 | 0.27 | 2.1 | 2.1 | 7.9 | -0.30 | 8.9 | 2.2 | 0.96 |
| MEN | | | | | | | | | | | | |
| Corn tortilla | 0.26 | 514.1 | 538.8 | 611.1 | 0.03 | 547.7 | 538.1 | 580.3 | 0.65* | 419.9 | 528.7 | 708.9 |
| Refined grains | 0.09 | 80.9 | 89.3 | 105.6 | 0.40* | 57.0 | 89.0 | 132.4 | 0.03 | 91.4 | 88.8 | 95.7 |
| Pastry | 0.14 | 56.4 | 58.6 | 65.3 | 0.26 | 44.1 | 63.6 | 73.4 | 0.21 | 45.6 | 62.2 | 71.8 |
| Salty snacks | 0.57* | 2.0 | 7.1 | 21.2 | 0.07 | 11.4 | 8.5 | 10.6 | 0.06 | 8.8 | 10.0 | 11.6 |
| Non-starchy vegetables | 0.13 | 96.4 | 105.5 | 151.9 | 0.48* | 60.4 | 110.9 | 186.8 | -0.15 | 134.5 | 111.5 | 108.8 |
| Starchy vegetables | 0.05 | 41.3 | 42.5 | 48.9 | 0.48* | 24.4 | 41.7 | 68.1 | -0.14 | 48.2 | 44.6 | 40.2 |
| Fried starches | 0.25 | 39.9 | 51.4 | 70.5 | 0.43* | 29.7 | 46.5 | 87.7 | -0.11 | 66.6 | 50.1 | 45.8 |
| Fruits | -0.03 | 297.4 | 230.9 | 267.5 | 0.48* | 117.1 | 271.3 | 415.6 | 0.03 | 253.7 | 239.8 | 300.7 |
| Eggs | 0.31 | 35.6 | 47.0 | 53.7 | 0.25 | 39.2 | 42.2 | 55.7 | 0.38* | 31.2 | 42.2 | 62.2 |
| Poultry | -0.04 | 23.2 | 18.4 | 20.3 | 0.38* | 13.3 | 19.0 | 30.2 | 0.10 | 16.5 | 19.5 | 25.6 |
| Red meat and pork | 0.20 | 11.1 | 17.8 | 21.3 | 0.33 | 11.0 | 14.3 | 25.5 | -0.29 | 23.3 | 14.8 | 12.5 |
| Processed meats | 0.15 | 14.5 | 15.4 | 19.2 | 0.42* | 6.2 | 16.0 | 27.7 | 0.07 | 13.4 | 17.9 | 17.8 |
| Giblets | 0.14 | 11.6 | 17.3 | 18.5 | 0.37* | 7.4 | 16.0 | 24.6 | -0.03 | 13.8 | 16.8 | 16.8 |
| Fish | 0.29 | 7.5 | 17.1 | 34.2 | 0.05 | 20.5 | 22.0 | 16.2 | -0.06 | 24.8 | 15.3 | 19.1 |
| Fried meats | 0.49* | 13.2 | 24.5 | 38.4 | 0.26 | 19.5 | 23.0 | 34.4 | 0.04 | 25.3 | 26.4 | 24.6 |
| Dairy | 0.06 | 57.7 | 68.2 | 89.3 | 0.39* | 34.6 | 62.8 | 120.9 | -0.29 | 92.2 | 64.2 | 59.9 |
| Beans | -0.09 | 147.8 | 132.7 | 121.2 | 0.05 | 126.4 | 130.4 | 145.4 | 0.65* | 74.7 | 109.5 | 214.0 |
| Oils/fats | 0.18 | 11.1 | 16.5 | 17.3 | 0.43* | 7.0 | 15.0 | 23.5 | 0.04 | 13.5 | 15.6 | 15.8 |
| Sugar added to coffee | -0.07 | 11.6 | 12.3 | 10.9 | -0.05 | 11.6 | 12.0 | 11.1 | 0.39* | 7.1 | 12.2 | 15.2 |
| Sweets | 0.51* | 2.4 | 5.1 | 14.3 | 0.23 | 4.9 | 6.0 | 11.1 | -0.19 | 10.3 | 5.8 | 5.8 |
| Alcohol | 0.45* | 9.2 | 49.8 | 150.2 | -0.19 | 123.4 | 46.4 | 38.3 | 0.09 | 53.4 | 63.9 | 91.8 |
| Sugar-sweetened beverages | 0.11 | 367.5 | 450.5 | 413.6 | 0.39* | 233.6 | 427.8 | 581.6 | 0.21 | 347.3 | 385.8 | 496.2 |
| Low-energy drinks^2^ | 0.68* | 71.1 | 216.2 | 373.4 | 0.04 | 225.8 | 237.3 | 195.0 | -0.02 | 236.7 | 203.4 | 220.2 |
| Packaged soup | -0.21 | 20.5 | 12.5 | 9.3 | 0.39* | 4.5 | 10.6 | 27.9 | -0.02 | 14.1 | 16.7 | 11.5 |
| Traditional Guatemalan foods^3^ | 0.37* | 27.9 | 49.5 | 76.9 | 0.18 | 41.8 | 52.3 | 61.3 | 0.14 | 44.5 | 49.1 | 60.8 |
| Transitional foods^4^ | 0.39* | 3.5 | 8.2 | 16.2 | 0.27 | 4.1 | 8.6 | 15.7 | -0.30 | 17.5 | 6.5 | 4.4 |
| 1. Mean intake of food groups (g) across tertiles.  2. Sparkling/soda water and coconut water.  3. Tamales and tacos.  4. Pizza and hamburgers.  Whole grains and nuts were excluded from analyses due to low (<10%) consumption. | | | | | | | | | | | | |

Abbreviations: INCAP, Institute of Nutrition for Central America and Panama; PCA, principal component analysis; T, tertile.
